# Factors Related to COVID-19 Vaccine Uptake in Black American Communities

**DOI:** 10.1093/geroni/igab046.892

**Published:** 2021-12-17

**Authors:** Julene Johnson, Orlando Harris, Carl V Hill, Peter Lichtenberg, Sahru Keiser, Thi Tran, Tam Perry, Elena Portacolone

**Affiliations:** 1 University California San Francisco, San Francisco, California, United States; 2 University of California, San Francisco, San Francisco, California, United States; 3 Alzheimer's Association, Chicago, Illinois, United States; 4 Wayne State University, Detroit, Michigan, United States; 5 UCSF/ San Francisco, UCSF Institute of Health and Aging/ San Francisco, California, United States; 6 University of California, Berkeley, California, United States

## Abstract

Black/African Americans represent 13% of the population, yet account for more than 24% of COVID-19 deaths. Emerging evidence indicates that Black Americans are receiving COVID-19 vaccines at lower rates than whites. However, there is minimal information about why vaccination rates are lower. To address this gap, we examined the effects of the COVID-19 pandemic among Black Americans, with an emphasis on understanding trust and vaccine uptake. Data were collected between July and September 2020 using 8 virtual focus groups in Detroit, MI and San Francisco Bay Area, CA with 33 older Black Americans and 11 caregivers of older Black Americans with cognitive impairment. Inductive/deductive content analysis was used to identify themes. The first theme pointed to a sense of feeling abandoned by healthcare providers and the government at local and state levels, which exacerbated uncertainty and fear about the vaccine and in general. The second theme emphasized a sense of deep distrust towards healthcare providers and the government, especially during the pandemic. The third theme pointed to a reluctance in receiving the vaccine because of distrust of pharmaceutical companies and the government, as well as misinformation and the rapid speed of vaccine development. These findings suggest that underlying systemic issues need to be addressed immediately to accelerate vaccine uptake among older Black Americans. New initiatives are needed to foster trust and address abandonment by healthcare and government systems. In addition, public health campaigns with reliable information about the COVID-19 vaccine are needed.

